# Remote Dielectric Sensing to Assess Residual Pulmonary Congestion Following Percutaneous Mitral Valve Repair

**DOI:** 10.3390/medicina58091292

**Published:** 2022-09-16

**Authors:** Teruhiko Imamura, Shuhei Tanaka, Hiroshi Ueno, Koichiro Kinugawa

**Affiliations:** Second Department of Internal Medicine, University of Toyama, Toyama 930-8555, Japan

**Keywords:** valvular disease, heart failure, lung fluid, mitral regurgitation, hemodynamics, MitraClip

## Abstract

*Background and Objectives*: Percutaneous mitral valve repair using a MitraClip system is an established therapeutic strategy to treat severe mitral regurgitation, which is recommended by guidelines in Europe and in the United States, whereas residual mitral regurgitation is associated with mortality and morbidity. Accurate assessment of residual mitral regurgitation is crucial for risk stratification and further adequate intervention, whereas its quantification has technical limitations due to “double” regurgitation that is often encountered following valve clipping. Remote dielectric sensing (ReDS^TM^) is a non-invasive electromagnetic-based technology to quantify lung fluid levels and might be a promising tool to assess the impact of residual mitral regurgitation following MitraClip. *Materials and Methods*: Following MitraClip, ReDS values measurements and right heart catheterization were performed and correlated. *Results*: We had 13 patients (median 74 years, 7 men) who underwent successful MitraClip. According to the visual estimation, eight patients had none or mild regurgitation, and five patients had moderate regurgitation. ReDS values were distributed widely between 16% and 33%, irrespective of the severity of regurgitation. ReDS values had a moderate correlation with invasively measured pulmonary artery wedge pressure (r = 0.73, *p* = 0.004). *Conclusions:* ReDS value might be a promising tool to assess residual pulmonary congestion following MitraClip, irrespective of the visually estimated severity of residual mitral regurgitation.

## 1. Introduction

Functional severe mitral regurgitation is associated with mortality and morbidity in patients with chronic heart failure. Percutaneous edge-to-edge mitral valve repair using MitraClip system (Abbott Laboratories, Abbott Park, IL, USA) has recently been introduced with favorable post-procedural clinical outcomes [1]. However, residual mitral regurgitation, some of which remains clinically significant, poses a potential challenge in achieving therapeutic success [2,3].

Transthoracic echocardiography is an established tool to quantify the degree of mitral regurgitation. However, accurate quantification of residual mitral regurgitation following MitraClip is challenging due to a “double” regurgitant jet, which is often encountered due to iatrogenic partial mitral valve approximating [4]. Following MitraClip, two regurgitant jets are often observed. This type of regurgitation is challenging to accurately quantify by echocardiography.

**Figure 1:**
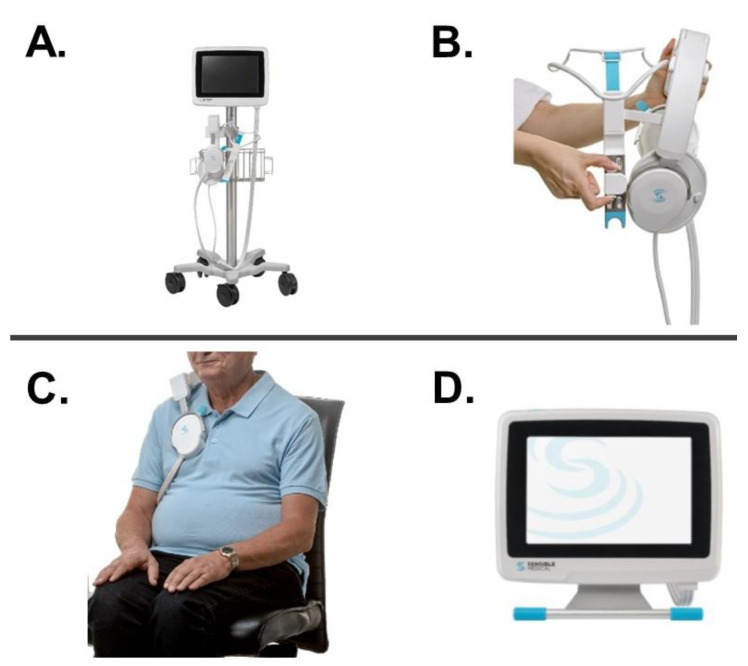
ReDS system consists of sensors and monitor (**A**). Low-power electromagnetic signals are emitted between the two sensors that are embedded in a wearable device (**B**). The sensors are worn at sitting position under natural breathing (**C**). The estimation of lung fluid volume (%), which is displayed on the monitor (**D**), takes 45 s. Sensible Medical Innovations Ltd. approved the use of this material. ReDS, remote dielectric sensing.

Remote dielectric sensing (ReDS^TM^, Sensible Medical Innovations Ltd., Netanya, Israel) is a recently introduced electromagnetic-based technology, which quantifies lung fluid levels (Figure 1) [5]. ReDS estimates the percentage of lung fluid level and quantifies pulmonary congestion. ReDS employs low-power electromagnetic signals emitted between the two sensors embedded in a wearable device. The measurement takes only 45 s and provides lung fluid content as a percentage of lung fluid volume (%) [6].

The technology might have a possibility to assess the impact of post-MitraClip residual mitral regurgitation on left atrial loading and resultant pulmonary congestion. If mitral regurgitation is appropriately treated by MitraClip without residual mitral regurgitation, ReDS values should theoretically decrease considerably. On the contrary, if residual mitral regurgitation is considerable, ReDS values should theoretically remain high due to persistently elevated left atrial pressures.

In this proof-of-concept study, we investigated the association between ReDS values and invasively measured pulmonary artery wedge pressure in patients undergoing MitraClip to treat functional mitral regurgitation.

## 2. Methods

### 2.1. Participant Selection

Consecutive patients with advanced mitral regurgitation who received MitraClip procedures between November 2021 and June 2022 were included in this proof-of-concept prospective study, and no patients declined to participate. Patients received ReDS measurements just prior to MitraClip without any exclusion criteria. One week following MitraClip, patients received (1) transthoracic echocardiography, as well as (2) ReDS measurements, as detailed below, and (3) invasive right heart catheterization. ReDS values were blinded to the examiners of other tests. The institutional ethical review board approved the protocol, and all participants signed informed consent.

### 2.2. Transthoracic Echocardiography

Two-dimensional and Doppler echocardiography was performed and standard echocardiography data, including visual assessment of residual mitral regurgitation following MitraClip procedures, were obtained by the board-certified cardiologists. The severity of mitral regurgitation was graded as none, trace, mild, moderate, moderate to severe, and severe [7]. Quantification of residual mitral regurgitation was not performed given the difficulty in accurate assessment of double regurgitant jets [4].

### 2.3. ReDS Measurements

ReDS values were measured at a sitting position under normal breathing as detailed previously (Figure 1) [5]. ReDS employs low-power electromagnetic signals emitted between two sensors embedded in wearable devices and measures the lung fluid volume as a percentage with manufacturer-recommended normal range between 20% and 35% [6].

### 2.4. Invasive Hemodynamics Measurements

Invasive right heart catheterization was performed by board-certified cardiologists and standard hemodynamics data, including pulmonary artery wedge pressure (PAWP), were obtained according to the standard manner. All procedures were performed via jugular venous access. All intra-cardiac data were obtained at an end-expiratory timing. All the procedures were supervised and obtained data were reviewed by the senior heart failure specialist.

### 2.5. Statistical Analyses

Continuous variables are presented as median and interquartile ranges (IQRs). Categorical variables are presented as numbers and percentages. Association between ReDS values and PAWP was assessed by Pearson’s correlation coefficient. Trends in ReDS values before and after MitraClip procedure was assessed using Wilcoxon signed-rank test. All calculations were performed in SPSS Statistics 23.0 software (IBM Corp., Armonk, NY, USA), and two-sided *p* values less than 0.05 were considered significant.

## 3. Results

### 3.1. Baseline Characteristics Following MitraClip Procedures

A total of 13 patients underwent successful MitraClip for advanced mitral regurgitation (Table 1). The median age was 74 (IQR 65, 81) years old and seven were males. Most of them (85%) had functional mitral regurgitation.

Following MitraClip, median left ventricular end-diastolic diameter was 55 (IQR 50, 78) mm and median left ventricular ejection fraction was 45% (IQR 31%, 53%). According to the visual estimation, eight (62%) patients had none or mild residual mitral regurgitation, and five (38%) patients had moderate mitral regurgitation. Median PAWP was 13 (IQR 10, 18) mmHg and median plasma B-type natriuretic peptide was 163 (IQR 100, 224) pg/mL. ReDS values were distributed widely between 16% and 33% with a median value of 28%.

### 3.2. Association between ReDS Values and PAWP

**Figure 2:**
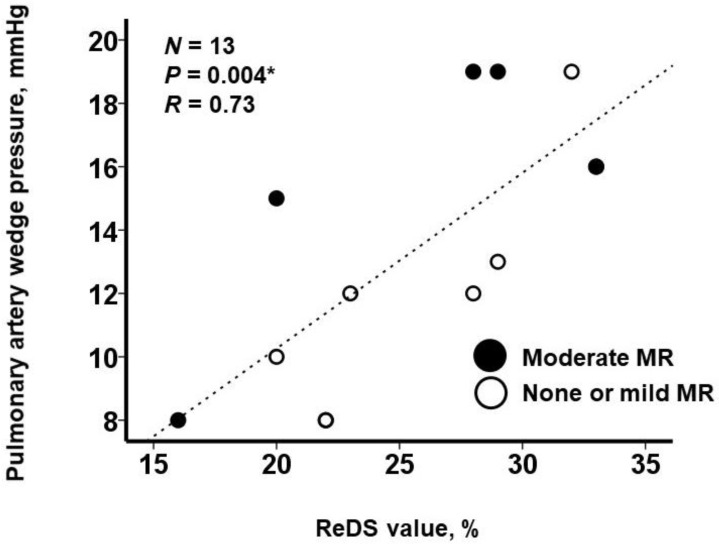
Correlation between ReDS values and PAWP among those receiving MitraClip procedures. MR, mitral regurgitation. White dots indicate none or mild MR, and black dots indicate moderate MR. * *p* < 0.05 by Pearson’s correlation coefficient.

ReDS values and PAWP had a moderate correlation (r = 0.73, *p* = 0.004; Figure 2). ReDS values are distributed widely, irrespective of the visually estimated severity of mitral regurgitation, i.e., none or mild (white dots) or moderate (black dots). Some patients had relatively higher ReDS values despite visually estimated none or mild mitral regurgitation.

### 3.3. Trend in ReDS Values before and after MitraClip Procedure

**Figure 3:**
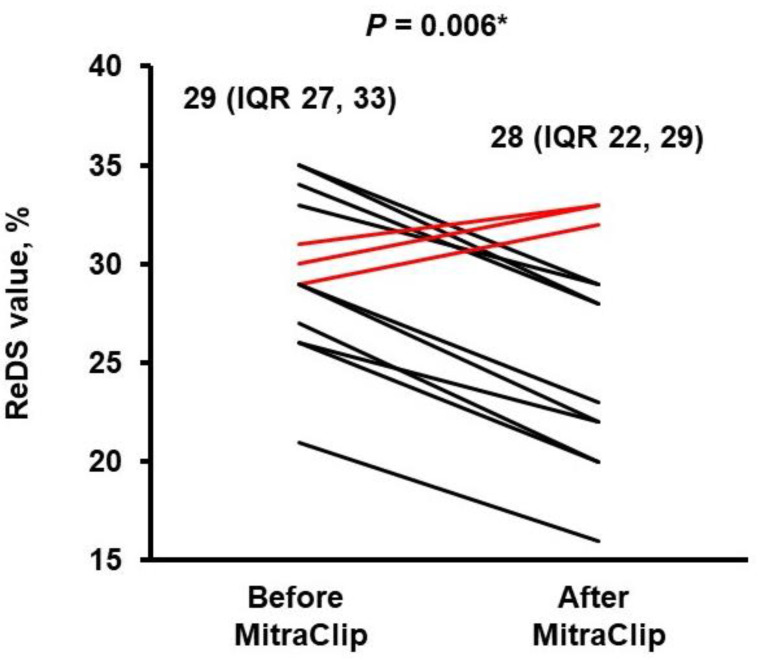
Trend in ReDS values before and after MitraClip.* *p* < 0.05 by Wilcoxon signed-rank test.

ReDS values prior to MitraClip was 29% (IQR 27%, 33%) on median, which decreased significantly following MitraClip (*p* = 0.006; Figure 3). However, ReDS values increased despite MitraClip in three patients (red bars in Figure 3). Their visually estimated residual mitral regurgitation were mild and moderate.

## 4. Discussion

In this proof-of-concept preliminary study, we demonstrated that ReDS values had a moderate correlation with invasively measured PAWP in patients following MitraClip, irrespective of the visually estimated severity of residual mitral regurgitation. Of note, some patients had relatively higher ReDS values despite visually estimated none or mild mitral regurgitation. ReDS values decreased following MitraClip in most of the patients, except for three patients.

MitraClip is an established therapeutic tool to treat severe mitral regurgitation [1], whereas residual mitral regurgitation remains a possibility for some patients, leading to persistent heart failure symptomology [2,3]. Given the nature of the double regurgitant jet, accurate quantification is challenging; accurate PISA cannot be drawn and estimation of elliptic mitral valve in the volumetric method is inaccurate [4]. Of note, its impact on pulmonary congestion needs accurate assessment by non-imaging modalities given the described challenges.

ReDS system has been demonstrated to be mildly or moderately correlated with other modalities, including high-resolution computed tomography [8,9] and invasively measured PAWP [10,11]. ReDS-guided heart failure management seems to have benefit in assessing congestion status [12]. In this study, we demonstrated that ReDS values had a moderate correlation with invasively measured PAWP among those undergoing MitraClip.

Our institute performed invasive right heart catheterization prior to index discharge following MitraClip during a specific study period, but it is not routinely performed in all institutes, as it is an invasive procedure and requires the appropriate resources. ReDS might be a promising alternative to invasive right heart catheterization to assess the impact of residual mitral regurgitation upon pulmonary congestion following MitraClip. As we observed, pulmonary congestion would decrease following MitraClip (black bars in Figure 3) if mitral regurgitation is appropriately treated. Persistently elevated ReDS values despite MitraClip (red bars in Figure 3) might indicate the existence of residual mitral regurgitation, procedure-related mitral stenosis, or peri-procedural volume overload.

Of note, the impact of residual mitral regurgitation might be sometimes underestimated by visual assessment alone. Even visually estimated mild mitral regurgitation might be associated with persistently elevated ReDS values, as we observed.

Thus, we highly recommend ReDS measurements for all candidates of MitraClip, irrespective of the visually estimated severity of residual mitral regurgitation. Further studies are warranted to validate the clinical implication of ReDS-guided management of residual pulmonary congestion following MitraClip.

### Limitations

This is a proof-of-concept preliminary study consisting of a small sample size that limited statistical implication. ReDS has been approved to be commercially available in Japan in June 2022, and further multi-center larger-scale studies are warranted to validate our findings. Moreover, the pharmacoeconomic and feasibility of utilizing ReDS for daily practice in standard institutes, by accounting for the cost of this device, should be validated. The association of ReDS values with other modalities among those receiving MitraClip remains unknown. ReDS values and PAWP were not extremely high and their association among those with decompensated heart failure following MitraClip procedure remains unknown. The prognostic impact of pulmonary congestion due to residual mitral regurgitation, which was indirectly assessed by ReDS technology in this study, remains a topic needing further investigation.

## 5. Conclusions

ReDS system might be a promising tool to assess the impact of residual mitral regurgitation upon pulmonary congestion in patients receiving MitraClip beyond the visual estimation of the severity of residual mitral regurgitation.

## Figures and Tables

**Table 1:** Baseline characteristics.

	*n* = 13
Demographics	
Age, years	74 (65, 81)
Male sex	7 (54%)
Body mass index, kg/m^2^	1.53 (1.41, 1.67)
Heart failure admission within one year	7 (54%)
Types of mitral regurgitation	
Degenerative	2 (15%)
Functional	11 (85%)
Comorbidity	
Hypertension	6 (46%)
Dyslipidemia	5 (39%)
Diabetes mellitus	4 (31%)
Atrial fibrillation	6 (46%)
History of stroke	2 (15%)
Chronic obstructive pulmonary disease	1 (8%)
Hemodialysis	1 (8%)
Echocardiography	
Left ventricular end-diastolic diameter, mm	55 (50, 78)
Left atrial diameter, mm	60 (52, 66)
Left ventricular ejection fraction, %	45 (31, 53)
Residual mitral regurgitation	
None or mild	8 (62%)
Moderate	5 (38%)
Hemodynamics	
Systolic blood pressure, mmHg	86 (84, 89)
Heart rate, bpm	68 (64, 72)
Mean right atrial pressure, mmHg	5 (4, 6)
Mean pulmonary artery pressure, mmHg	15 (14, 20)
Pulmonary artery wedge pressure, mmHg	13 (10, 18)
Cardiac index, L/min/m^2^	2.74 (2.40, 3.36)
Laboratory data	
Hemoglobin, g/dL	11.7 (10.2, 13.2)
Serum sodium, mEq/L	137 (136, 138)
Serum potassium, mEq/L	4.0 (3.9, 4.3)
Serum creatinine, mg/dL	1.48 (1.05, 1.77)
Plasma B-type natriuretic peptide, pg/mL	163 (100, 224)
Medications	
Beta-blockers	4 (31%)
Renin angiotensin system inhibitors	9 (70%)
Mineralocorticoid receptor antagonists	3 (23%)
Diuretics	4 (31%)
Remote dielectric sensing, %	28 (25, 29)

Continuous variables are stated as median and interquartile, and categorical variables are stated as number and percentage.

## Data Availability

Data are available upon reasonable requests.
